# Comparison of significance level at the true location using two linkage approaches: LODPAL and GENEFINDER

**DOI:** 10.1186/1471-2156-4-S1-S46

**Published:** 2003-12-31

**Authors:** Fang-Chi Hsu, Jacqueline B Hetmanski, Lan Li, Diane Markakis, Kevin Jacobs, Yin Yao Shugart

**Affiliations:** 1 Section on Biostatics, Department of Public Health Sciences, Wake Forest University School of Medicine, Winston-Salem, North Carolina, USA; 2 Department of Epidemiology, Bloomberg School of Public Health, Johns Hopkins University, Baltimore, Maryland, USA; 3 Division of Genetic Epidemiology, Department of Epidemiology and Biostatistics, Case Western Reserve University, Cleveland, Ohio, USA; 4 Division of Statistical Genetics and Bioinformatics, The OPAL Group, Cleveland, Ohio, USA

## Abstract

**Background:**

We compare two new software packages for linkage analysis, LODPAL and GENEFINDER. Both allow for covariate adjustment. Replicates 1 to 3 of Genetic Analysis Workshop 13 simulated data sets were used for the analyses. We described the results of searching for evidence of loci contributing to a simulated quantitative trait related to systolic blood pressure (SBP). Individuals with SBP greater than 130 mm Hg were defined as affected individuals, and all others as unaffected. Total cholesterol was treated as a covariate.

**Results:**

Using LODPAL, the power of detecting one of the three major genes related to SBP is 44.4% when a LOD score of 1 is used as the cut-off point. The power of GENEFINDER is lower than that of LODPAL. It is 22.2%.

**Conclusions:**

Based on the limited comparison, LODPAL provided the more reasonable power to detect linkage compared to GENEFINDER. After adjusting for the total cholesterol covariate, the current version of both programs appeared to give a high number of false positives.

## Background

There has been great interest in developing linkage analysis methods that allow for the adjustment of covariates, because this type of analysis can potentially allow us greater power to detect genetic effects after adjusting traits for the possible effect of covariates. The object of this study is to compare the performance of two genetic model-free linkage analysis software packages: LODPAL and GENEFINDER. Below we present some brief theoretical background on the two statistical methods implemented in LODPAL and GENEFINDER.

### LODPAL

LODPAL is an affected-relative-pair analysis method using a conditional-logistic model that allows covariates to adjust the relative risks associated with sharing alleles identity by descent (IBD) [1]. Goddard et al. [2] modified the two-parameter method originally described by Olson [1] by assuming a mathematical relationship between the two model parameters λ_1 _and λ_2_, where λ_1 _is the relative risk for a pair of relatives that shares exactly one allele IBD and λ_2 _is the relative risk for a pair of relatives that shares two alleles IBD. Olson's original method requires two additional parameters for each covariate, while the new method needs only one parameter by using the relationship λ_2 _= 3.634 × λ_1 _- 2.634. This idea of parameter reduction was based on the work described by Whittmore and Tu [3], in which they showed that a minimum-maximum one-parameter ASP LOD score had better power for most genetic models than traditional two-parameter models when assuming a genetic model "approximately half way between a recessive and a dominant mode of inheritance".

### GENEFINDER

Liang et al. [5] developed a multipoint linkage mapping approach for estimating the location of a trait locus using affected sibling pairs. This method makes an assumption that there is no more than one trait locus in the chromosomal region. It has been implemented in the software called GENEFINDER. The primary statistics are the number of alleles shared IBD from multiple markers. The model can be expressed as

E (*S*(*t*) | Φ) = 1 + (1-2θ_*t*,τ_)^2^(E(*S*(τ) | Φ) - 1)

= 1 + (1-2θ_*t*,τ_)^2 ^× *C*,

where *S(t) *is the number of alleles shared IBD at an arbitrary locus *t *in the chromosomal region, Φ is the event of affected siblings, θ is the recombination fraction between locus *t *and the unobserved trait locus τ, and *C *is defined as (E(*S*(τ) | Φ) - 1), which is the effect of the unobserved trait locus as characterized by the excessive IBD sharing due to the linkage to an unobserved trait locus. Using the generalized estimating equation (GEE) procedure, we can estimate the parameters of interest, τ and *C*, and their confidence intervals, directly. An interesting feature of this approach is that one can test the null hypothesis of no linkage to this region by testing *C *= 0, which follows a χ^2 ^distribution with 1 df. Furthermore, this GEE approach has been extended to incorporate the linkage evidence from unlinked regions [6] and to incorporate covariate information [7]. When incorporating covariate data, the model can be expressed as

E(*S(t) *| x ∈ l,Φ) = 1 + (1 -2θ_*t*,τ_)^2^(E(*S*(τ) | x ∈ *l*, Φ) - 1)

= 1 + (1 - 2θ_*t*,τ_)^2 ^× *C*_*l*_,

where *x *is the discrete covariate information, *l*(= 0, 1, 2) is the value of this covariate, and *C*_*l *_is defined as (E(*S*(τ) | Φ) - 1) for the pairs with a covariate coded as 0, 1, and 2, respectively. Similarly, we can estimate τ, *C*_*l*_, and their confidence intervals. One can test the null hypothesis of no linkage to this region by testing *C*_0 _= *C*_1 _= *C*_2 _= 0, which follows a χ^2 ^distribution with 3 df.

## Methods

### Selection of phenotype and preliminary analysis

The details of the simulation data set of the Genetic Analysis Workshop 13 (GAW13) were described elsewhere [8]. We were interested in examining the power to detect linkage using covariate-based linkage analysis methods using Replicates 1 to 3 of the GAW13 data set. Particularly, we selected systolic blood pressure (SBP) as our trait of interest and dichotomized the trait by coding individuals with SBP over 130 mm Hg as affected and all others as unaffected. (We first considered using SBP over 140 mm Hg as the threshold. However, there were not enough affected pairs for the purpose of methodology evaluation with this threshold.) For the sake of simplicity, we have only used the phenotypic information from the first exam for each individual. Presumably, at this earlier date, fewer individuals had been medicated due to high blood pressure and/or high cholesterol levels.

We identified 330 families with a total of 4692 individuals. The data contained the following relative pairs: parent-offspring (5840), sib-sib (2798), grandparent-child (6220), avuncular (2175), half-sib (77) and cousin (1747). For LODPAL, 175 relative pairs from Replicate 1, 177 relative pairs from Replicate 2, and 199 relative pairs from Replicate 3 with informative allele-sharing information were included in the analyses. The covariate "total cholesterol" was treated as a continuous variable.

Since the current version of GENEFINDER only allows for affected sibling pairs, the information from pedigrees with affected relative pairs other than siblings will be discarded. For Replicate 1, 109 affected sibling pairs from 50 families were used; for Replicate 2, 107 affected sibling pairs from 60 families were used; and for Replicate 3, 112 affected sibling pairs from 65 families were used. Since GENEFINDER can only handle discrete covariates, we dichotomized the total cholesterol based on a cut point at 200 mg/dl. Therefore, individuals with total cholesterol greater than 200 mg/dl were considered to have high cholesterol. Based on this classification, there were 48 sibling pairs both with low cholesterol (LL), 44 pairs with only one low cholesterol (HL), and 17 pairs both with high cholesterol (HH) for Replicate 1. For Replicate 2, the numbers of sibling pairs used for analysis were 37, 40, and 30, respectively. For Replicate 3, the numbers were 53, 41, and 18, respectively. In our analysis, covariates for these three scenarios were coded as *l *= 0, 1, and 2, respectively.

### Linkage analysis

We performed a series of multipoint LODPAL analyses on Replicates 1 to 3 of the GAW13 data using SAGE version 4.3 [9]. We also completed a series of multipoint linkage analyses using GENEFINDER version 1.0 [10]. All 22 chromosomes from the three replicates were analyzed. The results generated on the chromosomes containing true loci related to SBP are then compared to those generated by GENEFINDER.

## Results

**Figure 1:**
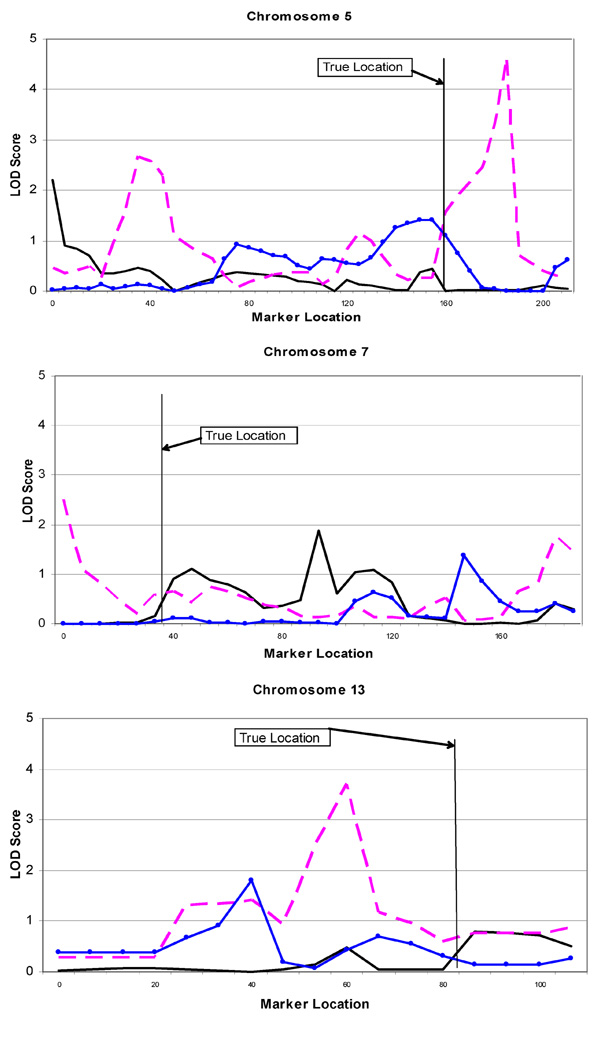
Genome scan results of LODPAL for SBP on chromosomes 5, 7, and 13, where the true quantitative trait loci located (black, Replicate 1; pink, Replicate 2; blue, Replicate 3).

For the LODPAL analysis, we chose to use a LOD score of 1 as the threshold for suggestive findings. Using this threshold, we detected a total of one true locus on chromosome 7 (LOD = 1.87, *p*-value = 0.0013) in Replicate 1, two true loci on chromosome 5 (LOD = 4.629, *p*-value = 0.0029) and 13 (LOD = 3.699, *p*-value = 0.009) in Replicate 2, and one true locus on chromosome 5 (LOD = 1.87, *p*-value = 0.009) in Replicate 3 (see Figure 1). We defined the "true-positive signal" to be a marker giving a LOD score equal or greater than 1 and located less than 40 cM from a true locus. The false-positive rates are 0.059 for Replicate 1, 0.177 for Replicate 2, and 0.133 for Replicate 3.

For the GENEFINDER analysis, without adjusting for covariate, the significance of linkage evidence was detected on chromosome 7 in Replicate 2 only (*p*-value = 0.049). After adjusting for covariate, it was detected on chromosome 5 in Replicate 1 (*p*-value = 0.036) and chromosome 7 in Replicate 2 (*p*-value = 0.016). However, the estimates of the locations of trait loci were not close to the true ones on chromosomes 5 (at 176.08 cM) and 7 (at 47.49 cM). Relatively, GENEFINDER located a trait locus closer to the true location of quantitative trait locus B35 (at 85.16 cM) on chromosome 13 in Replicates 1 and 2. Further, the false-positive rates are much higher after adjusting for the covariate when compared to those without adjusting for the covariate (0.263 vs. 0.053 in Replicate 1; 0.316 vs. 0.105 in Replicate 2; 0.579 vs. 0.000 in Replicate 3); see Table 1.

## Discussion and Conclusions

Our analysis based on GAW13 simulated data showed that the power to detect linkage in a complex trait is reasonable using the LODPAL approach and the addition of covariates increases the power to a certain extent (data not shown). However, it is important to estimate empirical *p*-values when using the LODPAL approach, especially when the pedigree structure includes several pair types with overlapping individuals [J.M. Olson, personal communication]. The analysis based on the same simulation data showed that the power to detect linkage for a complex trait using the GENEFINDER approach is less powerful and the addition of covariate also increases power.

Although our comparison of LODPAL and GENEFINDER is limited, there are still some points worth mentioning. First, the addition of covariates increased the possibility of getting a false-positive result in our analyses. Because total cholesterol is a real risk factor for SBP, the models without adjustment are less powerful when compared with those with adjustment. Second, the appropriate selection of a cut point for total cholesterol for GENEFINDER may have had an impact on our power to detect linkage. An advantage of using LODPAL is that one does not have to dichotomize covariates, because it naturally models continuous covariates. Third, the cut point for SBP is 130 mm Hg, which is lower than the normal cut-off of 140 mm Hg. This may partly explain why we observed lower power, but higher false positive rates. Fourth, unlike LODPAL, GENEFINDER is designed to be used after previous evidence of linkage has been found [5] because it can further hone in on a chromosomal region of interest. This strategy can also help reduce the false positives. In this paper, we performed the GENEFINDER analysis for all the chromosomes. In other studies, we can focus on those regions where the linkage evidence has been identified.

Furthermore, we stress the advantages and disadvantages of these two methods. While both methods are model-free and allow for the adjustment of covariates, LODPAL also allows for continuous covariates not just categorical ones. GENEFINDER, on the other hand, only allows for categorical covariates, which presents a limitation. However, GENEFINDER can be used to test the location of an unobserved trait locus and also provide its 95% confidence interval. The other advantage is that GENEFINDER, a multipoint approach, can also be used to test the null hypothesis of no linkage using a test with 1 df without adjusting for a covariate and with 3 df after adjusting for a covariate. In LODPAL, as indicated by Goddard et al. [2], the distribution of likelihood ratio statistic (LRS) for one parameter is a 50:50 mixture of a point mass at 0 and a χ^2 ^distribution with 1 df. Additional covariates gives an LRS with a distribution that is a 50:50 mixture of a χ^2 ^with k df and a χ^2 ^with (k+1) df. Also note that GENEFINDER is sensitive to the initial values of the estimates. Therefore, exploring different initial values to ensure that the solution reaches a global maximum is highly recommended because it can help reach the convergent criteria.

Finally, we would like to note that using the programs we evaluated required dichotomization of a quantitative trait and therefore may cause a noticeable amount of power loss. On the other hand, dichotomizing a trait such as blood pressure is not an uncommon practice in real studies. Therefore, it is of interest to examine the power to detect linkage under a situation where the design is less than optimal. Since we only performed analyses on three replicates (due to the limited time, computational resources, and the relatively variable state of newly developed software), it is difficult to give solid guidelines to future users. However, we would like to caution the users with respect to the large number of false positives produced by both software packages.

## Figures and Tables

**Table 1:** Summarized results of GENEFINDER analysis

	**Replicate 1**				**Replicate 2**				**Replicate 3**			
	
	**Without covariate**		**With covariate**		**Without covariate**		**With covariate**		**Without covariate**		**With covariate**	
	
**Chr**	**LE^A^**	**CI^B^**	**LE**	**CI**	**LE**	**CI**	**LE**	**CI**	**LE**	**CI**	**LE**	**CI**
1	n^C^		192.4	(181.7, 203.1)	251.7	(237.3, 260.1)	248.5	(237.0, 260.0)	238.6	(221.5, 255.7)	245.1*	(235.4, 254.7)
2	n		156	(141.8, 170.1)	227.9	(192.3, 263.6)	285.2	(275.5, 295.0)	216.3	(198.4, 234.1)	224.3*	(215.4, 233.2)
3	n		31.1	(18.6, 43.6)	67.9	(52.9, 83.0)	199.5	(188.0, 211.0)	151.1	(115.5, 186.6)	150.2	(140.8, 159.7)
4	176.1	(158.3, 193.9)	196.7**	(191.2, 202.3)	84.5	(65.7, 103.3)	92.7	(77.7, 107.6)	85.2	(76.5, 93.9)	86.4***	(81.7, 91.1)
5	75.2	(46.2, 104.2)	16.2*	(9.3, 23.2)	83.6	(50.4, 116.8)	85	(72.7, 97.3)	n		56.4	(49.3, 63.6)
6	46.2**	(40.9, 51.5)	53.0*	(48.3, 57.6)	94.1*	(80.9, 107.2)	90.2***	(80.9, 99.5)	52.9	(42.4, 63.4)	50.7	(40.4, 61.1)
7	n		110.2	(94.3, 126.0)	69.2*	(59.7, 78.6)	79.5*	(72.6, 86.3)	n		83.47	(71.6, 95.4)
8	n		142.2	(133.0, 51.3)	114.5	(92.1, 137.0)	114.4	(97.5, 131.3)	2	(0, 120.0)	54.65	(40.4, 68.9)
9	26.3	(14.4, 38.2)	24.7	(10.1, 39.2)	138.4*	(128.1, 148.6)	138.1*	(128.1. 148.0)	16.6	(2.5, 30.7)	22	(9.8, 34.3)
10	187.9	(175.4, 200.4)	151.4*	(144.3, 158.6)	47.9	(35.9, 59.8)	49.2	(38.7, 59.7)	n		173.9*	(165.0, 182.9)
11	5.8	(0, 38.5)	38.1	(28.3, 48.0)	53.4	(33.3, 73.6)	57.5	(47.0, 68.0)	n		52.7*	(44.7, 60.7)
12	146.4	(120.0, 172.8)	72	(62.5, 81.5)	n		69.7***	(63.35, 76.1)	79.4	(60.9, 97.8)	33.7*	(24.0, 43.3)
13	63.1	(16.2, 100.0)	94.4	(84.2, 104.6)	87.5	(72.8, 102.2)	67	(58.1, 76.0)	n		112.3	(99.1, 125.5)
14	118.2	(72.9, 163.6)	107.4***	(103.4, 111.4)	n		79.4	(57.2, 101.7)	n		106.9	(91.5, 122.3)
15	123	(105.0, 140.9)	112.1	(107.1, 117.2)	n		82.8*	(74.0, 91.6)	92.8	(54.7, 130.8)	104.3	(94.0, 114.5)
16	46.7	(27.1, 66.2)	48.1	(34.5, 61.7)	92.8	(63.1, 122.5)	120.1*	(110.9, 129.2)	124.1	(0, 297.3)	116.7	(98.6, 134.8)
17	137.5	(115.2, 159.8)	134.5	(122.1, 146.9)	118.6	(97.9, 139.2)	80.4	(62.8, 97.9)	n		57.6*	(51.1, 64.1)
18	n		23.2	(11.5, 34.8)	25.1	(3.3, 46.8)	24.3	(3.5, 45.0)	n		11.5**	(5.7, 17.3)
19	n		72.0**	(59.4, 84.7)	14.8	(0.1, 29.4)	29	(14.7, 43.2)	–^D^		68.4***	(63.2, 73.7)
20	56.5	(36.8, 76.3)	88.1	(80.5, 95.7)	n		95.7	(73.3, 118.0)	36.2	(20.0, 52.3)	39.7*	(32.0, 47.4)
21	–		47.9	(27.6, 68.2)	n		38.9	(23.5, 54.3)	n		42.1*	(37.1, 47.2)
22	–		40	(25.9, 54.1)	17.7	(0, 66.5)	37.6**	(33.2, 42.1)	38.9	(12.5, 65.3)	9.9	(0, 25.4)

^A^LE, location estimate. ^B^CI, 95% confidence interval. ^C^n, converge to the minimum not the maximum. ^D^_, not convergent. *p-value < 0.05; **p-value < 0.005; ***p-value < 0.0005
